# Hypoxia Induces a Metabolic Shift and Enhances the Stemness and Expansion of Cochlear Spiral Ganglion Stem/Progenitor Cells

**DOI:** 10.1155/2015/359537

**Published:** 2015-07-05

**Authors:** Hsin-Chien Chen, Jen-Tin Lee, Cheng-Ping Shih, Ting-Ting Chao, Huey-Kang Sytwu, Shiue-Li Li, Mei-Cho Fang, Hang-Kang Chen, Yi-Chun Lin, Chao-Yin Kuo, Chih-Hung Wang

**Affiliations:** ^1^Department of Otolaryngology-Head and Neck Surgery, Tri-Service General Hospital, National Defense Medical Center, No. 325, Section 2, Cheng-Kung Road, Taipei 114, Taiwan; ^2^Department of Otolaryngology, Auditory Medical Center, Cheng Hsin General Hospital, No. 45, Cheng Hsin Street, Taipei 112, Taiwan; ^3^Graduate Institute of Medical Sciences, National Defense Medical Center, No. 161, Section 6, Minquan East Road, Taipei 114, Taiwan; ^4^Medical Research Center, Cardinal Tien Hospital, No. 362, Zhongzheng Road, Xindian District, New Taipei City 23148, Taiwan; ^5^Graduate Institute of Microbiology and Immunology, National Defense Medical Center, No. 161, Section 6, Minquan East Road, Taipei 114, Taiwan; ^6^Laboratory Animal Center, National Defense Medical Center, No. 161, Section 6, Minquan East Road, Taipei 114, Taiwan

## Abstract

Previously, we demonstrated that hypoxia (1% O_2_) enhances stemness markers and expands the cell numbers of cochlear stem/progenitor cells (SPCs). In this study, we further investigated the long-term effect of hypoxia on stemness and the bioenergetic status of cochlear spiral ganglion SPCs cultured at low oxygen tensions. Spiral ganglion SPCs were obtained from postnatal day 1 CBA/CaJ mouse pups. The measurement of oxygen consumption rate, extracellular acidification rate (ECAR), and intracellular adenosine triphosphate levels corresponding to 20% and 5% oxygen concentrations was determined using a Seahorse XF extracellular flux analyzer. After low oxygen tension cultivation for 21 days, the mean size of the hypoxia-expanded neurospheres was significantly increased at 5% O_2_; this correlated with high-level expression of hypoxia-inducible factor-1 alpha (Hif-1*α*), proliferating cell nuclear antigen (PCNA), cyclin D1, Abcg2, nestin, and Nanog proteins but downregulated expression of p27 compared to that in a normoxic condition. Low oxygen tension cultivation tended to increase the side population fraction, with a significant difference found at 5% O_2_ compared to that at 20% O_2_. In addition, hypoxia induced a metabolic energy shift of SPCs toward higher basal ECARs and higher maximum mitochondrial respiratory capacity but lower proton leak than under normoxia, where the SPC metabolism was switched toward glycolysis in long-term hypoxic cultivation.

## 1. Introduction

Unlike cochlear hair cells in the peripheral pathway, spiral ganglion neurons play a central role by sending sound information from the cochlea to the brain for hearing transduction [1]. However, similar to various nerve cells in the body, the spiral ganglion may undergo degeneration or damage through aging, noise exposure, chemical toxins, disease, and genetic disorders and usually cannot be replaced after being destroyed. Such neurodegeneration not only affects the integrity of the auditory system, but also may limit the functional benefit of cochlear implants [2, 3]. The recent emergence of stem cell-based medicine has the potential to revolutionize the study of neurodegenerative diseases. It has therefore motivated intense investigation on developing stem cell therapy as a new therapeutic strategy, for example, through the transplantation of stem cells into the inner ear for hearing restoration [4].

Postnatal adult stem cells currently have attracted intense interest in stem cell research due to the lower possibility of teratoma formation, accessible donor cells, and tissue-specific cell fate determination. Since a very limited number of isolated adult stem cells may exist in some specific organs, such as the eye and cochlea [5, 6], it is very important to develop strategies to expand sufficient populations of adult stem cells for present investigation and future application.

Previously, we reported that short-term hypoxic cultivation would benefit from expanding cochlear stem/progenitor cells and maintaining their stemness markers through activation of hypoxia-inducible factor-1 alpha (Hif-1*α*) [7]. Similar benefits of mild hypoxia had also been observed in cultivated human neural stem cells by enhancing their expansion and multipotency [8]. Since the metabolic state is likely to influence the maintenance of the stem cell population and determine the cell fate of stem cells [9–11], it would be interesting to elucidate whether the metabolic signature is correlated with hypoxia-related stemness status and differentiation potential.

We hypothesized that the metabolism of cochlear spiral ganglion stem/progenitor cells (SPCs) in hypoxia differs from that in a normoxic condition. To test this, we cultivated cochlear spiral ganglion SPCs at different oxygen tensions to delineate their metabolic status and stemness properties; this represents the first such report in the literature.

## 2. Materials and Methods

### 2.1. Isolation and Culture of Cochlear Spiral Ganglion SPCs

The cochlear spiral ganglion SPCs were isolated using the method described in our previous report [7]. Briefly, the cochlear modiolus housing spiral ganglion cells and neuronal fibers were harvested from the cochleae of postnatal day 1 (P1) CBA/CaJ mouse pups. Using enzymatic and mechanical methods, these newly dissociated spiral ganglion-derived cells were plated in a noncoated T25 flask (Nunc) at 37°C in a 5% CO_2_ atmosphere serum-free DMEM/F12 supplemented with penicillin-G, 20 ng/mL of epidermal growth factor (EGF, R&D), 10 ng/mL of basic fibroblast growth factor (b-FGF, R&D), 50 ng/mL of insulin growth factor (IGF, R&D), and N2 and B27 (GIBCO) supplements on the first day* in vitro* (DIV). The medium was changed every 3 days. After 7 DIV, primary spheres were observed. For secondary spheres, primary spheres were collected followed by mechanical dissociation with a Pasteur pipette and 0.05% trypsin. The dissociated primary spheres were maintained in a T25 flask for secondary sphere formation. To allow for continuous expansion, half of this medium was replaced every day and cultures were passaged every seventh day.

### 2.2. Immunocytochemistry

For immunocytochemistry staining, secondary spheres were either transferred to 24-well plates with coverslips and cultured in DMEM/F12 medium supplemented with 10% fetal bovine serum (FBS) overnight or prepared using a cytospin at 1,200 rpm for 5 min. The attached spheres were fixed in phosphate-buffered saline- (PBS-) buffered 4% paraformaldehyde and 2% sucrose, washed three times with PBS, permeabilized with 3% bovine serum albumin in PBS containing 0.3% Triton X-100, and blocked with 5% normal goat serum. Coverslips were incubated with mouse monoclonal anti-nestin antibody (1 : 500; Abcam) and rabbit polyclonal anti-Nanog antibody (1 : 200; Abcam) at 4°C overnight. After three washes with PBS, coverslips were incubated with fluorescein isothiocyanate- (FITC-) or tetramethyl rhodamine isocyanate- (TRITC-) conjugated secondary antibody (1 : 200; Thermo Fisher Scientific) to reveal the cell markers and stained with 4′,6-diamidino-2-phenylindole, dihydrochloride (DAPI; 0.66 mg/mL in PBS; Molecular Probes) for visualization of nuclei. Coverslips were mounted onto slides and examined under an epifluorescence microscope.

### 2.3. Cell Differentiation

Secondary spheres were cultured under adherent conditions in 24-well plates filled with DMEM and 10% FBS. The medium was changed every second day. After 96 h, differentiated cells were analyzed by immunocytochemistry. We used mouse monoclonal antibody to *β*-III tubulin (1 : 500; Thermo) and rabbit polyclonal antibody to glial fibrillary acidic protein (GFAP; 1 : 500; Abcam).

### 2.4. Bromodeoxyuridine (BrdU) Incorporation

Detection of BrdU incorporation in DNA-synthesizing cells was performed by adding 10 mM of BrdU (Sigma) to the secondary sphere. After a 72-h incubation period, spheres were plated onto coverslips in a 24-well plate containing DMEM/F12 and 10% FBS. After 24 h of cell seeding, coverslips were fixed and incubated in 2N HCl for 30 min at 37°C. Immunodetection of BrdU was performed using a monoclonal antibody against BrdU (1 : 500; Sigma). Fluorescence TRITC-tagged secondary antibody (1 : 200) was employed for visualization.

### 2.5. Hypoxia Incubation

Hypoxic culture conditions were continuously applied to newly isolated spiral ganglion cells or dissociated primary spheres in a N_2_/CO_2_ multigas incubator (APM-50D, Astec, Japan) by setting two different low oxygen tension (1% and 5% O_2_) conditions and in a 5% CO_2_ atmosphere at 37°C for the indicated time interval. The control group was exposed to a normoxic incubator at 37°C with 95% air and 5% CO_2_. The medium was changed every 3 to 4 days.

### 2.6. Cell Proliferation Assay

Dissociated primary spheres were seeded in a 96-well plate (5 × 10^3^ cells/well) and exposed to 1%, 5%, and 20% O_2_ conditions in a 5% CO_2_/37°C incubator for the indicated time interval. The medium was changed every 4 days. To determine cell proliferation, 10% WST-1 (Roche) agent was added to cell suspension in each well and incubated for 4 h. The reaction was catalyzed by a mitochondrial reductase in active cells, and the amount of formazan dye could be quantified by measuring the absorbance at 450 nm using Bio-Rad enzyme-linked immunosorbent assay (ELISA) reader to calculate the optical density (OD) values (A450 nm–A655 nm). Statistical analysis was determined using the Student's *t*-test, with *P* < 0.05 considered significant.

### 2.7. Western Blot Analysis

Primary spheres were seeded in six-well plates and cultured for 96 h at different oxygen concentrations (1%, 5%, and 20% O_2_), respectively. Total cell lysates were prepared by lysing the spheres in a sample buffer (66 mM Tris-HCl, pH 7.4, 2% sodium dodecyl sulfate [SDS]) at 90°C. Lysates containing equal amounts of protein were loaded and separated on 8% SDS polyacrylamide gels. After electrophoresis, the gels were transferred to polyvinylidene difluoride (PVDF) membranes (Millipore), blocked with 5% skimmed milk in TBST (0.2 M Tris-base, 1.37 M NaCl, and 0.1% Tween 20), and probed with the indicated primary antibody at 4°C overnight. After washing three times with TBST, the membranes were then incubated with a peroxidase-conjugated secondary antibody for 1 h at room temperature and washed with TBST. The immunoreactive bands were stained using a light emitting nonradioactive method (ECL; Millipore). The specific primary antibody includes mouse anti-Hif-1*α* monoclonal antibody (1 : 500; Santa Cruz), mouse anti-proliferating cell nuclear antigen (PCNA) monoclonal antibody (1 : 1,000; BD Bioscience), mouse anti-cyclin D1 monoclonal antibody (1 : 1,000; Santa Crus), mouse anti-Abcg2 monoclonal antibody (1 : 1,000; Millipore), mouse anti-nestin monoclonal antibody (1 : 1,000; Abcam), rabbit anti-Nanog polyclonal antibody (1 : 1,000; Abcam), mouse anti-p27 monoclonal antibody (1 : 1,000; Neo-Markers), and rabbit anti-actin polyclonal antibody (1 : 2,000; Chemicon).

### 2.8. Side Population (SP) Cell Analysis Using Hoechst 33342 Staining and Flow Cytometry

SP cell analysis was carried out using the method previously described [12]. Briefly, following different oxygen tension exposures for 96 h, newly isolated spiral ganglion cells (5 × 10^5^ cells/well) were dissociated and suspended in prewarmed medium at 37°C for 30 min. In the absence or presence of 1 *μ*M fumitremorgin C (FTC; Alexis Biochemicals), cells were incubated with 5 *μ*g/mL Hoechst 33342 (Sigma) at 37°C for 60 min, followed by washing with PBS. Samples were centrifuged and resuspended in cold PBS supplemented with 3% FBS (Biological Industries). Propidium iodide (PI; Sigma-Aldrich) was added at a final concentration of 2 *μ*g/mL to exclude dead cells. FACS was performed using the BD FACSAria flow cytometer (BD Biosciences). The Hoechst dye was excited with an ultraviolet (UV) laser at 355 nm. A live gate was defined on the FACS using Hoechst red and blue axes to exclude dead cells and debris. Flow cytometry using Hoechst 33342 dye exclusion as a guiding parameter can determine the boundary between SP and non-SP cells. After 10^5^ events were collected within the live gates, SP and non-SP cells were sorted and defined as Hoechst-low and Hoechst-bright cells, respectively.

### 2.9. Oxygen Consumption and the Extracellular Acidification Rate (ECAR)

The mitochondrial oxygen consumption rate (OCR) and ECAR were measured using a Seahorse Bioscience XF24 extracellular flux analyzer (Seahorse Bioscience). Before the day of the assay, the cartridge sensor was hydrated overnight with 1 mL Seahorse Bioscience XF24 Calibration Buffer at 37°C without CO_2_. On the day of the assay, SPCs were seeded in an XF24 Islet Capture Microplate and the growth medium was replaced with serum-free DMEM/F12 lacking sodium bicarbonate. Cells were then incubated at 37°C in a non-CO_2_ incubator for 1 h. OCR and ECAR values were monitored under basal condition and measured after the injection of oligomycin (1 *μ*M), FCCP (carbonyl cyanide p-trifluoromethoxyphenylhydrazone, 1 *μ*M), and antimycin A (1 *μ*M) to the well in succession. OCR and ECAR results were analyzed using the Seahorse XF-24 software. Every point represents an average of five different wells.

### 2.10. Determination of Intracellular ATP

For comparison of relative ATP levels between hypoxic and normoxic conditions, the ATP assay was conducted using the ATP Bioluminescence Assay Kit CLS II (Roche). Cells (2 × 10^5^) were lysed and centrifuged at 10,000 g for 60 s. The supernatant was reacted with luciferase reagent as instructed in the manufacturer's protocol.

### 2.11. Statistical Analysis

Statistical analysis was performed using a two-tailed Student's *t*-test. Results are expressed as means ± standard error of the mean (SEM). Differences were considered significant at *P* < 0.05.

## 3. Results and Discussion

### 3.1. Identification and Characterization of Cochlear Spiral Ganglion SPCs

**Figure 1:**
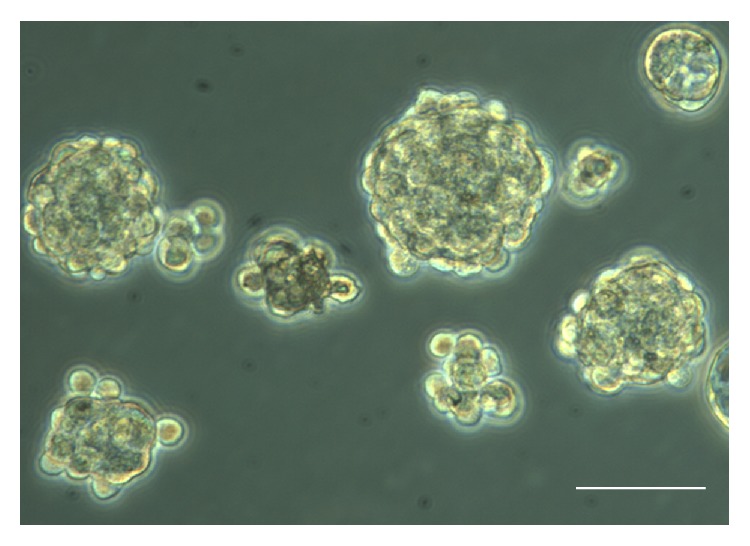
Newly isolated spiral ganglion SPCs derived from mice cochleae of P1 neonates formed spheres at day 7 in the ultralow plate containing serum-free medium. Scale bar = 75 *μ*m.

**Figure 2:**
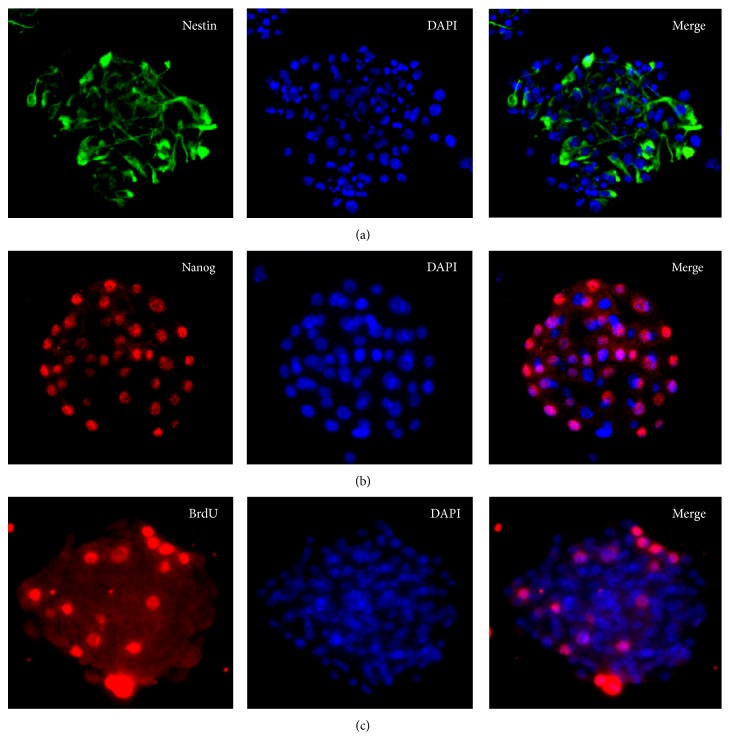
SPC markers and BrdU incorporation were revealed by immunocytochemistry. (a) Secondary spheres derived from spiral ganglion SPCs express nestin, an intermediate filament protein predominantly expressed by neural stem cells. (b) Nanog expression was observed in the nuclei of the majority of sphere-forming cells. (c) BrdU incorporation was detected in the nuclei of spheres. The blue-fluorescent DAPI nucleic acid stain for visualization of nuclei. Original magnification ×200.

Primary spheres derived from the cochlear spiral ganglion with a solid morphological population were produced after 7 DIV (Figure 1). These spheres were further dissociated and cultured in ultralow-attachment 6-well plates for another 7 days to generate secondary spheres. We used the secondary spheres to identify the stem cell markers and investigate their proliferative ability. Immunostaining confirmed the expression of stem cell markers nestin (Figure 2(a)) and Nanog (Figure 2(b)) in these spheres with BrdU incorporation (Figure 2(c)), implying that the spheres possess stem-like and self-renewal properties.

**Figure 3:**
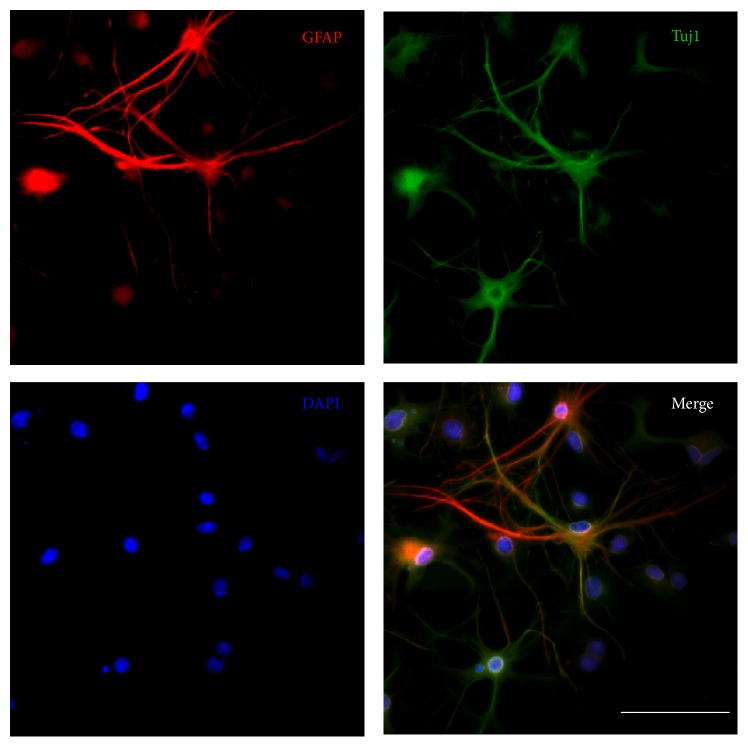
Induction of differentiation in spiral ganglion spheres. Secondary spheres were cultured in adherent conditions with the presence of 10% FBS in DMEM/F12 medium for 96 h. Immunocytochemistry reveals that some of them were found to differentiate into glial-like cells by expressing GFAP (red). Concurrently some sphere-forming cells differentiated into neural cells by expressing neuron-specific *β*III tubulin (Tuj1, green). A small population of cells was immunostained by both GFAP and Tuj1 (merge). Scale bar = 100 *μ*m.

To investigate the potency of spiral ganglion SPCs, secondary spheres were cultured in adherent condition with 10% FBS containing DMEM/F12 for 96 h to allow for differentiation.  Figure 3 demonstrates that the differentiated cells from spiral ganglion SPCs were able to express glial cell protein GFAP or neural marker *β*III-tubulin. In addition, a small population of cells expressed both neural and glial cell proteins simultaneously. These results suggest that SPCs derived from the P1 mouse spiral ganglion are capable of proliferating and possess multipotency to differentiate into neuron and glial cells; this finding is supported in other reports [13] and is consistent with our previous research, which showed that cochlear SPCs from a postnatal cochlea retain characteristic stem-like and pluripotent differentiation potential [7].

The successful induction of cochlear spiral ganglion SPCs into neuron and glial cells may have several effects. First, it implies that spiral ganglion-derived SPCs are ready to adopt a spiral ganglion cell fate without the need for further genomic manipulation of donor cells. Second, in our study, spiral ganglion SPCs were proved to differentiate easily into neuronal lineages. Finally, replacement of damaged spiral ganglion neurons is feasible either via direct transplantation of SPCs into the inner ear alone or via being combined with cochlear implant surgery. Recently, Zhang et al. [3] identified mouse inner ear statoacoustic ganglion-derived neural progenitors that could be successfully induced into spiral ganglion-like cells by nerve growth factor after implantation into the adult mammalian inner ear. By transplantation of otic neural progenitor cells that were derived from human embryonic stem cells (hESCs) and able to differentiate* in vitro* into hair-cell-like cells and auditory neurons, Chen et al. [4] demonstrated a restoration of auditory evoked responses from an auditory neuropathy model. These studies have provided a new promising approach to restoring lost hearing by neural progenitor cell replacement therapy.

### 3.2. Hypoxia Enhances the Sphere Formation and Proliferation of Cochlear Spiral Ganglion SPCs

**Figure 4:**
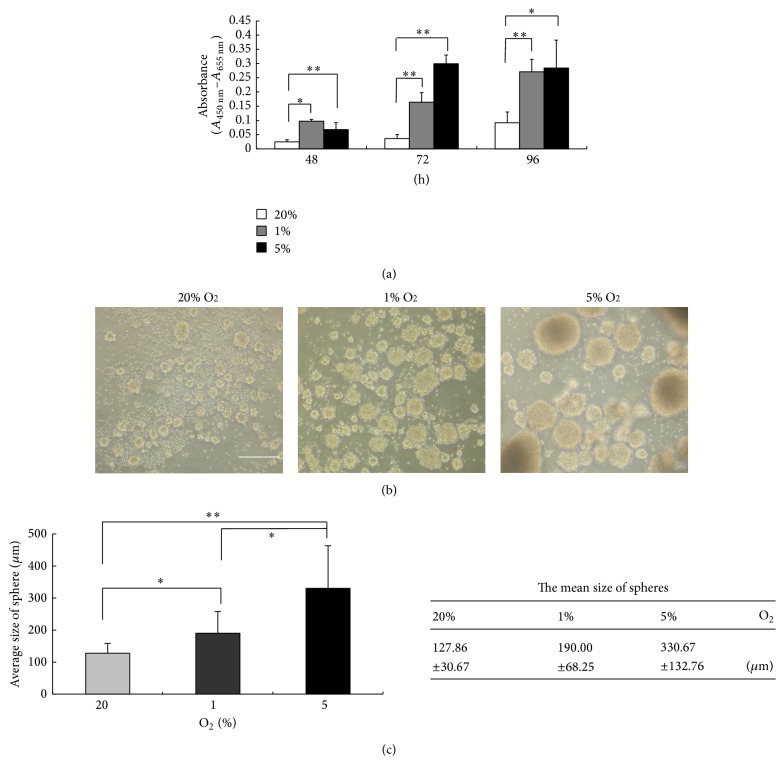
(a) The effects of different oxygen tensions on the proliferation of cochlear spiral SPCs were examined using the WST-1 test. The proliferative capacities in the hypoxic groups significantly increased compared with the normoxic group at each time point. (b) Morphological changes of the spheres in each oxygen tension group were observed on 21 DIV. (c) Quantitative analysis of sphere sizes generated from single-cell cultures of SPCs undergoing different oxygen tension culturing was carried out on 21 DIV. Results are expressed as mean ± SEM with *n* = 5 for each bar. Scale bar = 300 *μ*m; *∗* indicates *P* < 0.05; *∗∗* indicates *P* < 0.01.

To evaluate the hypoxia effect on cell proliferation, newly dissociated SPCs following different oxygen tension exposures for 48, 72, and 96 h were compared by WST-1 assay. The results indicated that low oxygen tensions significantly enhanced the proliferation of SPCs compared with normoxic conditions at each indicated time point (Figure 4(a)). In addition, newly dissociated single primary spiral ganglion cells underwent culturing at 1%, 5%, or 20% O_2_; sphere formation could be observed in each oxygen tension group after 7 DIV, and the spheres were found to increase in size following long-turn culturing. On 21 DIV, a significant sphere size difference was observed between the hypoxia and the normoxia groups (Figures 4(b) and 4(c)). Furthermore, in hypoxic conditions, the sphere size at 5% O_2_ was even significantly larger than that at 1% O_2_ (330.67 ± 132.76 *μ*m versus 190.00 ± 68.25 *μ*m, *P* < 0.05; Figure 4(c)). These results indicate that low oxygen tensions benefit expanding cochlear spiral ganglion SPCs* in vitro*, which is in agreement with previous studies demonstrating that the culture of human stem cells over a physiological range of low oxygen tensions improves cell growth and expends their lifespan [14, 15]. Moreover, exposure of mammalian cells to 20% O_2_ was shown to result in DNA damage [16, 17], whereas low oxygen tension improved the genetic stability of cultured human mesenchymal stem cells [14].

### 3.3. Hypoxia Upregulated the Expressions of Proliferation and Stemness Markers of Cochlear Spiral Ganglion SPCs

**Figure 5:**
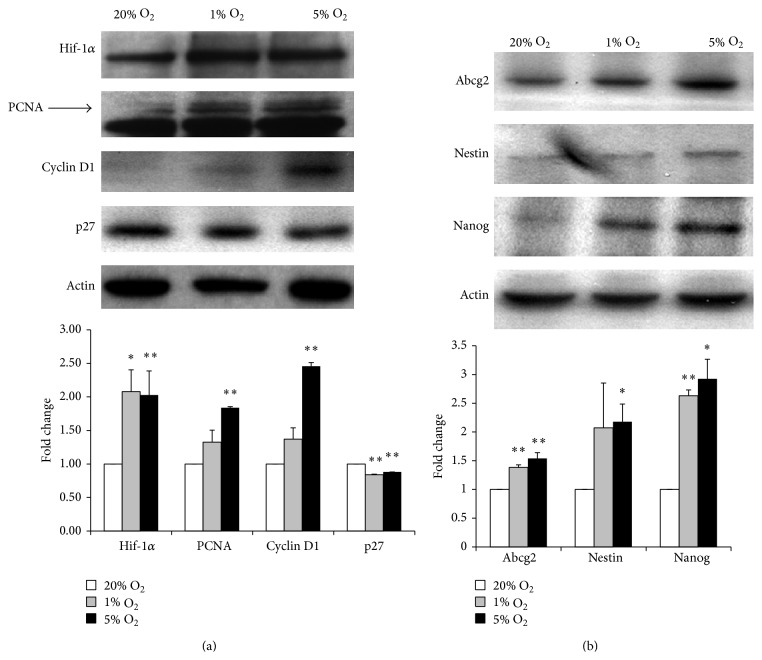
Western blot-based comparison of proliferation (a) and stemness (b) related proteins on cochlear spiral ganglion SPCs when exposed to different oxygen tensions for 4 days. Densitometric analysis of the western blot is reported in the histogram and shown in the lower panels. For the comparison, the expression levels of each protein (mean ± SEM, *n* = 3) were normalized to control actin protein levels and expressed as a fold change of the cells cultured at 20% O_2_. *∗* indicates *P* < 0.05; *∗∗* indicates *P* < 0.01.

Previously, we demonstrated that cochlear SPCs cultured at 1% O_2_ for 24 h enhanced their growth through Hif-1*α* [7]. However, the effect of different low oxygen tensions on the protein expression profile of proliferation and stemness of cochlear spiral ganglion SPCs has not yet been demonstrated. Given that Hif-1*α* is regulated mainly by oxygen tension through the oxygen-dependent degradation of its *α* subunit, we expected that low oxygen tension would be involved in controlling the cell cycle, cellular proliferation, or apoptosis by initiating a gene expression program through the Hif-1*α*. As shown in the results, the protein level of Hif-1*α* was increased at 1% and 5% O_2_ compared to that at 20% O_2_ (Figure 5(a)). Meanwhile, cyclin D1 and PCNA protein levels were also increased in low oxygen tensions, concomitant with a decreased protein level of p27^Kip1^ (p27) (Figure 5(a)).

G1/S-specific cyclin D1 functions as a regulatory subunit of cyclin-dependent kinases (CDKs), whose activity is required for cell cycle G1/S transition. Overexpression of cdk4/cyclin D1 was found to increase the generation of basal progenitors and shorten the G1 of neural stem cells; thus, it can be used to increase progenitor expansion [18]. Regarding the significance of PCNA protein expression, Walters and Zuo showed that mouse cochleae at P1 and P2 were able to exhibit PCNA in hair cells and supporting cells, whereas by P5, PCNA was no longer detected in these cells [19]. This implies that persistence of PCNA in postnatal cochlear hair cells and supporting cells may retain some of the factors necessary for cell cycle entry. Therefore, PCNA can be used to mark cell proliferation and to identify a population of progenitor cells [20]. The role of the cell cycle inhibitor p27 in maintaining the stemness of hESCs has been demonstrated by showing the low expression level of p27 in hESCs, but this will lead to a G1 phase arrest, cell differentiation, and consequent loss of self-renewal ability when p27 is overexpressed [21]. Taken together, in this study, we found that hypoxia activates and amplifies cochlear spiral ganglion SPCs, reflected by the elevated expression of cyclin D1 and PCNA and decreased expression of p27. Concurrently, stemness-related protein expression, including Abcg2, nestin, and Nanog, was markedly induced at low oxygen tension compared to that at standard 20% O_2_, with the most abundant protein level found at 5% O_2_ (Figure 5(b)).

Hif-1*α* is believed to play a role of pivotal link between oxygen availability in the cell and several important processes such as energy metabolism, angiogenesis, and cell proliferation and viability [14, 22]. The impact of hypoxia involving Hif-1*α* signaling on SPC self-renewal, differentiation, maturation, and homing in various* in vitro* and* in vivo* settings was shown in our previous study [7] and other published literatures [23–25]. We demonstrated again in this study that Hif-1*α* is activated and associated with enhanced proliferation and stemness-related gene expression in cochlear spiral ganglion SPCs when exposed to low oxygen tensions.

### 3.4. Hypoxia Enhanced the SP Distribution

**Figure 6:**
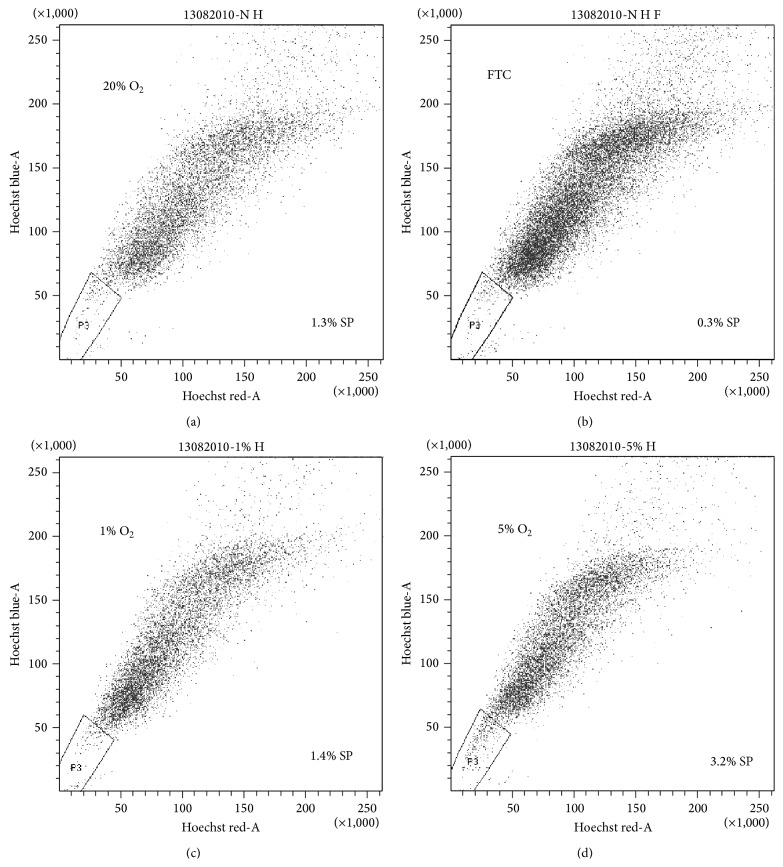
Isolation of an SP cell population was conducted on SPCs following different oxygen tension exposures for 4 days. Cells were stained with Hoechst 33342 dye either alone or in the presence of FTC and analyzed by flow cytometry. SP cells were gated and are shown as the percentage of total SPCs when cultured at 1%, 5%, or 20% O_2_. Hoechst dye efflux was markedly diminished in FTC-treated SPCs.

Isolation of SP cells has been recognized as a useful technique for the identification of cochlear SPCs [12, 26]. As shown in Figure 6, SPCs cultured at 5% O_2_ prompted a prominent increase in the percentage of SP cells to 3.2%, whereas at 20% O_2_, SPCs contained only 1.3% SP cells. That the obtained SP fraction markedly diminished through the addition of FTC helped to verify the specificity of the SP subpopulation obtained from the spiral ganglion SPCs. These results support low oxygen tension culture as a strategy for efficiently expanding cochlear SPCs or spiral ganglion SPCs without losing stem cell properties such as proliferation and self-renewal.

Although most organisms require O_2_ for survival, as this is the primary substrate for energy production in the cell, physiological O_2_ concentrations in developing embryos are generally lower (2–9% O_2_) than in ambient air (21% O_2_) [27]. In this study, we observed that low oxygen tensions drastically increased the number and size of sphere formation, as well as the SP fraction in cochlear spiral ganglion SPCs, indicating that* ex vivo* expansion of spiral ganglion SPCs under hypoxia may be practically applied for the large-scale production and maintenance of SPCs for stem cell-based replacement therapy. Given that low oxygen tensions maintain undifferentiated states of embryonic, hematopoietic, mesenchymal, and neural stem cell phenotypes and modulate proliferation and cell-fate commitment [28], it is reasonable to assume that a much lower oxygen tension than that of ambient air in some specific environments of the cochlea may be more suitable for stem or progenitor cell populations to reside. Meanwhile, a real stem cell niche in the adult cochlea needs to be explored further.

### 3.5. Hypoxia Shifts the Metabolic Pathway of Spiral Ganglion SPCs Predominantly toward Glycolysis

**Figure 7:**
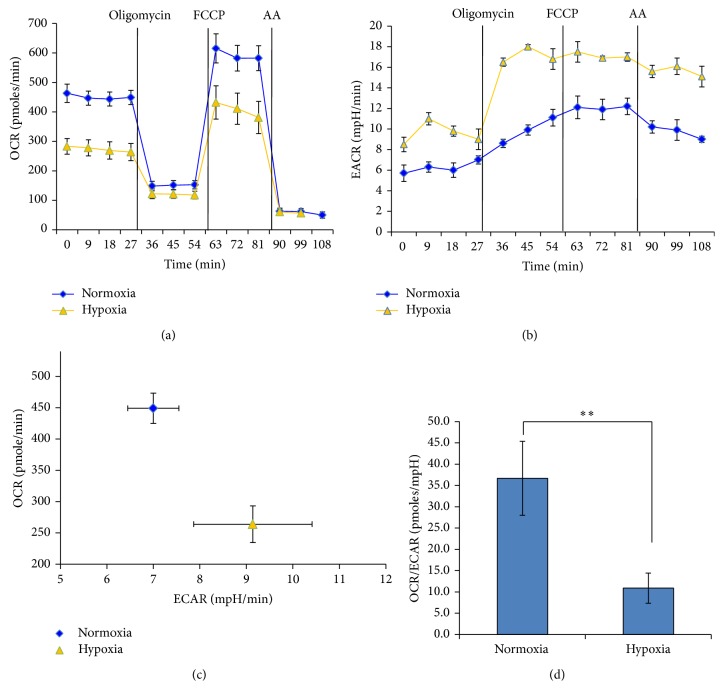
Cochlear spiral ganglion SPCs cultured in hypoxia conditions undergo a metabolic switch from oxidative phosphorylation to glycolysis. Real-time measurements (mean ± SEM, *n* = 5) of (a) the mitochondrial OCR (pMols/min) and (b) ECAR (mpH/min) of SPCs cultured in normoxia or hypoxia conditions were measured under basal condition and in response to the indicated mitochondrial inhibitors. The OCR was lower in hypoxia condition. (c) The basal OCR and ECAR values of normoxia- and hypoxia-cultured SPCs were plotted to illustrate the difference in cellular bioenergetics (mean ± SEM, *n* = 5). (d) OCR to ECAR ratios measured by the XF24 extracellular flux analyzer (mean ± SEM, *n* = 5) show a significant decrease in SPCs exposed to hypoxia, indicating a metabolic transition from mitochondrial oxidative phosphorylation to glycolysis. *∗∗* indicates *P* < 0.01; FCCP: carbonyl cyanide p-trifluoromethoxyphenylhydrazone; AA: antimycin A.

The relationship between mitochondrial metabolism and cell proliferation and stemness in spiral ganglion SPCs remains poorly understood. We further characterized the bioenergetics status change of SPCs at different oxygen tensions using Seahorse noninvasive technology [29]. We determined the mitochondrial OCR and ECAR, which represent the measurement of oxidative phosphorylation (OXPHOS) and glycolysis, respectively, from cells grown at either 20% or 5% O_2_ for 2 months. We showed that the basal OCR of SPCs was markedly low at 5% O_2_ compared with 20% O_2_ (Figure 7(a)). The addition of oligomycin, a natural antibiotic that inhibits F0/F1 ATPase (complex V), differentiates the ATP-linked respiration from the proton leak. Following oligomycin addition, the maximal respiratory rate was determined by subsequent addition of FCCP, an uncoupler that raises OCR to an extremely high level. Finally, injection of antimycin A inhibited the flux of electrons through complex III and thus determined the remaining OCR resulting from nonmitochondrial respiration, as no further oxygen was consumed at the cytochrome c oxidase.

As shown in Figure 7(b), the response of ECAR at each oxygen tension to mitochondrial toxicants reflected a concurrent glycolytic rate after mitochondrial perturbation. Following oligomycin treatment, the greater response of ECAR in hypoxia-cultured cells suggested that SPCs cultured under hypoxia are more sensitive to mitochondria perturbation than under normoxia. Another plausible explanation is that glycolysis is less efficient than OXPHOS in gathering energy from glucose; hypoxic SPCs therefore need to increase the rate of glucose uptake and glycolysis to meet its energy demands [30].

When SPC cells were cultured under hypoxia, OXPHOS levels decreased by at least 40%, while basal ECAR activity increased by 30% in relation to normoxia, indicating a metabolic pathway change from OXPHOS to glycolysis (Figure 7(c)). This was also reflected in the greatly reduced OCR to ECAR ratio when SPCs were cultured under hypoxia (Figure 7(d)). Such metabolic switch is likely attributable to the activation of Hif-1*α* signaling, because cells exposed to low oxygen levels that fall below a certain threshold would increase the amount of glycolytic enzymes and glucose transporters through the Hif-1*α* pathway [31, 32], while downregulating the enzymes driving mitochondrial metabolism [33]. Overexpression of Hif-1*α* in ESCs has also been shown to switch their energy production pathways from bivalent toward higher glycolytic activity [34].

### 3.6. Cochlear Spiral Ganglion SPCs Display Higher Maximal Mitochondrial Respiratory Capacity in Hypoxic Conditions

**Figure 8:**
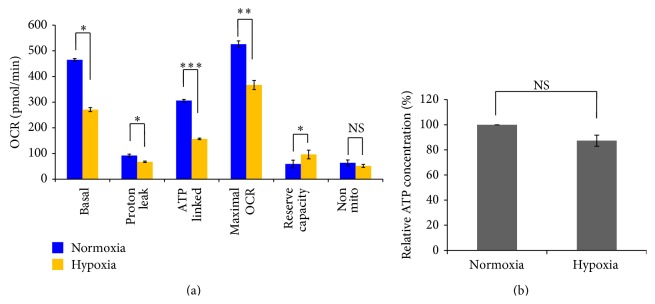
Measurement of mitochondrial function in cochlear spiral ganglion SPCs cultured in normoxic and hypoxic conditions. Results are expressed as mean ± SEM with *n* = 5 for each bar. NS indicates a non-significant difference; *∗* indicates *P* < 0.05; *∗∗* indicates *P* < 0.01; *∗∗∗* indicates *P* < 0.005.

Although cochlear spiral ganglion SPCs consumed O_2_ at a lower rate in hypoxia than in normoxia, as demonstrated in Figure 7(a), it was unclear what proportion of their maximal electron transport capacity was being utilized. The difference in mitochondrial function of SPCs cultured in normoxic and hypoxic conditions was defined by sequentially adding specific mitochondrial inhibitors, which allowed each component of the respiratory chain to be delineated. FCCP is a mitochondrial uncoupler (protonophore) that dissipates the mitochondrial membrane potential to stimulate maximal electron transport and O_2_ consumption [35]. As shown in Figure 8(a), the reserve capacity of cells grown in hypoxia was significantly greater than for normoxia, implying that hypoxia-cultured SPCs may have greater substantial capacity than those in normoxia in response to stress or pathologically relevant injury, increasing energy demands for the maintenance of organ function, cellular repair, or detoxification of reactive species [36]. In contrast, proton leak significantly decreased at a lower oxygen tension, suggesting that SPCs grown under hypoxia might have less mitochondrial membrane damage compared to normoxic culture. The decreased proton leak shown in hypoxia SPCs may imply reduced oxidative stress encountered in such a microenvironment. Together, these results support the view that a general description of stem cell metabolism usually involves increased glycolysis, limited oxidative metabolism, and resistance to oxidative damage [37].

To examine whether there is difference of ATP production between hypoxia and normoxia, the data showed that SPCs maintain equivalent levels of ATP when cultured in normoxia and hypoxia (Figure 8(b)). This suggests that SPCs cultured in hypoxia were able to upregulate glycolysis and maintain sufficient ATP levels following the inhibition of OCR by mitochondrial inhibitors.

## 4. Conclusions

In this study, we demonstrated that cochlear spiral ganglion SPCs from P1 neonates could be amplified by hypoxia, with subsequent differentiation into neurons and glia. The effect of low oxygen tension on spiral ganglion SPCs not only resulted in enhancing proliferation in the cell amount and diameter of neurospheres, but also increased the protein expression of cyclin D1 and PCNA, while at the same time suppressing the p27 level. Coupled with this is the upregulation of stemness-related marker proteins Abcg2, nestin, and Nanog. Within a new field of cellular bioenergetics investigation, we showed that SPCs grown at low oxygen tension have a significantly higher mitochondrial reserve capacity compared to that under normoxia. Hypoxia also significantly decreased their proton leak when SPCs were cultured in hypoxia. These data suggest a diminished oxidative stress encountered in a hypoxic microenvironment of spiral ganglion SPCs, along with a greater substantial capacity than normoxia in response to mitochondrial perturbation due to a higher maximum respiratory capacity. Unlike cells grown in normoxia, hypoxia-cultured SPCs shift their metabolic pathway predominantly to glycolysis but still maintain sufficient ATP levels as they would grow under normoxia. The upregulated Hif-1*α* shown in hypoxia-cultured SPCs indicated that the dominant glycolytic metabolism and cellular proliferation may be mediated through Hif-1*α* activation. These findings suggest a role of Hif-1*α* for spiral ganglion SPCs in response to hypoxia* in vitro* and suggest a possible mechanism for their enhanced proliferation under hypoxic conditions.
